# The association between glycated hemoglobin and intraocular inflammatory factors in patients with proliferative diabetic retinopathy

**DOI:** 10.1186/s40942-025-00732-y

**Published:** 2025-10-22

**Authors:** Jun Liu, Miao Yu, Jingyu Yan, Wei Chen, Pei Zhang, Yuchen Wang, Huijin Chen, Jinping Hu

**Affiliations:** 1 Ophthalmology Department, Peking University Third Hospital, No. 49 North Garden Road, Haidian District, Beijing, 100191 China; 2 Nursing Department, Peking University Third Hospital, Beijing, China

**Keywords:** Diabetic retinopathy, HbA1c, Inflammatory cytokines, Aqueous humor, Restricted cubic splines

## Abstract

**Purpose:**

This study explored the correlation between Hemoglobin A1c (HbA1c) and intraocular inflammatory cytokines in proliferative diabetic retinopathy (PDR) patients, aiming to provide evidence for PDR prevention and management.

**Methods:**

This was a cross-sectional study involving 100 patients and 100 eyes diagnosed with PDR. All patients were collected demographic characteristics, HbA1c, and history of chronic diseases at baseline. Aqueous humor samples of patients were obtained during the intraocular surgery. Then 10 cytokines were assessed using the Cytometric Beads Array (CBA) technique. The relationships between HbA1c and each inflammatory factor were further tested by restricted cubic splines (RCS) in the logistic model.

**Results:**

A U-shaped curve was found between IL-6 (interleukin-6) and HbA1c (*p* = 0.0001). HbA1c was negatively associated with IL-6 when below 8.31%. There was also a U-shaped exposure-response relationship between vascular endothelial growth factor (VEGF) and HbA1c (*p* = 0.0211), with the lowest VEGF levels observed at an HbA1c of 6.72%. A J-shaped curve was observed in the relationship between IL-8 (interleukin-8) and HbA1c (*p* = 0.0061). When HbA1c exceeded 8.23%, IL-8 exhibited a significant upward trend. Monocyte chemoattractant protein-1 (MCP-1) shows a positive linear relationship with HbA1c (*p* = 0.0036). No significant differences were found between other cytokines and HbA1c.

**Conclusions:**

Our study identified the correlation between HbA1c and intraocular inflammation in PDR, characterized by U-shaped relationships with IL-6 and VEGF, a J-shaped relationship with IL-8, and a linear relationship with MCP-1. These findings advocated for a nuanced approach to glycemic management. However, longitudinal studies are still needed to determine whether HbA1c fluctuations directly drive cytokine changes.

**Clinical trial registration number:**

Not applicable.

**Supplementary information:**

The online version contains supplementary material available at 10.1186/s40942-025-00732-y.

## Introduction

Diabetic retinopathy (DR) is a common and specific microvascular complication of diabetes and is the leading cause of visual loss in the elderly [1]. 146 million adults worldwide are estimated to suffer from DR [2]. With the progress of the disease, DR starts from the non-proliferative diabetic retinopathy (NPDR), and then develops to the PDR. Once it develops into PDR, patients may experience severe vision impairment due to vitreous hemorrhage or traction retinal detachment [3]. The prevention and control of DR is the key to protect vision and improve quality of life.

The complex and interrelated pathophysiological mechanisms, including increased production of inflammatory factors, underlie the development of DR triggered by Hyperglycemia [4]. Elevated cytokine levels induce leukocyte activation and migration, leading to capillary occlusion, retinal hypoxia, endothelial cell damage, and ultimately the breakdown of the blood-retinal barrier [5]. Increasing evidence shows that systemic inflammation is an intrinsic mechanism of occurrence and development of DR [6]. As the core mediators of immune and inflammatory regulatory networks, significant changes in cytokines show key value in physiological homeostasis monitoring and pathological mechanism analysis [7].

As known, VEGF is a core pathogenic driver of DR, triggering endothelial cell proliferation, migration, and vascular permeability [8]. IL-6 mediates insulin-induced vascular remodeling and enhances retinal permeability through VEGF upregulation [9, 10]. IL-8 and MCP-1 recruit immune cells (neutrophils/T cells and monocytes/lymphocytes), synergistically promoting angiogenic factor secretion in PDR [11]. Adhesion molecules intercellular cell adhesion molecule-1 (ICAM-1) [12] and cluster of differentiation 106 (CD106)/vascular cell adhesion molecule-1 (VCAM-1) [13] further amplify DR inflammation. IL-10 (interleukin-10), as an immunosuppressive cytokine, may counteract inflammatory ocular damage, with its elevation in diabetic eyes potentially reflecting a compensatory response to pro-inflammatory mediators [14, 15]. Meanwhile, C-X-C motif chemokine ligand 10 (CXCL10)/interferon gamma-induced protein 10 (IP-10)—a chemoattractant in Th1 responses, shows elevated levels in NPDR/diabetic macular oedema (DMO) aqueous humor alongside, while interferon-gamma (IFN-γ) and granulocyte colony-stimulating factor (G-CSF) levels are decreased, suggesting immune dysregulation—though the accuracy of these findings requires further validation [16]. Cytokine biomarkers may serve as critical tools for monitoring the onset and progression of DR. The precise quantification of intraocular biomarkers, such as inflammatory factors, contributes to the precise medical approach of DR [17].

HbA1c serves as the gold standard for evaluating the level of glycemic control and reflects the average blood glucose levels over the preceding two to three months. HbA1c is a critical predictor for the risk of developing DR; for each 1% increase in HbA1c, the risk of DR increases by 25% [18]. Also, a research shows that HbA1c is an independent risk factors for the development of PDR [6]. The International Diabetes Federation advises that the target for HbA1c should be less than 7% for most adults with diabetes. However, the rapid reduction of hyperglycemia may also be a risk factor for DR progression [19]. Personalized goals should be set considering factors such as age, overall health status, and the risk of hypoglycemia [20]. Therefore, the targets for HbA1c must carefully balance the risks and benefits, taking into account the progression of the disease and the specific clinical context of each individual.

Currently, most studies examine the relationship between HbA1c and DR using multifactor analysis methods [21–23]. Although these studies have provided some insights, research on determining the optimal HbA1c values to reduce the risk of DR is still relatively limited. To fill this gap, this study employs a statistical model known as RCS to explore the correlation between HbA1c levels and intraocular inflammatory cytokines in patients with PDR. This knowledge is crucial for the development of new therapeutic approaches, the formulation of preventive strategies, and the improvement of existing management plans. In short, this study attempts to provide more precise glycemic control targets for the prevention and management of DR, in order to reduce the risk of PDR.

## Methods

### Study design and participants

This study was a cross-sectional study approved by the Peking University Third Hospital Ethics Committee (2025-033-01), and all participants provided written informed consent. Patients waiting for intraocular surgery were recruited from Peking University Third Hospital Eye Center between February 2025 and March 2025.

The inclusion criteria for the sample were as follows: (a) aged ≥18 years; (b) diagnosed with PDR; and (c) had been scheduled for surgery within one week. The exclusion criteria were as follows: (a) diagnosed with type 1 diabetes mellitus; (b) underwent ophthalmic surgery or intravitreal injection in the last six months; (c) with renal failure or routine peritoneal dialysis; (d) with autoimmune disease, hematological disease, or malignancy; (e) applied corticosteroids systemically or locally within the last three months.

### HbA1c measurement

A 5 ml whole blood sample of each included patient was collected at baseline, a week before surgery. HbA1c analysis were performed using EASYCELL 206A1. The analytical precision of this system demonstrated coefficients of variation of 1.2% at 6% HbA1c and 1.1% at 11.4% HbA1c.

### Aqueous sampling, processing, and analysis

Following standard aseptic preparation involving preoperative disinfection and placement of sterile surgical drapes, undiluted aqueous humor specimens (0.05–0.1 mL) were obtained from participants. Aseptic anterior chamber paracentesis was performed at the corneal limbus using a 30-gauge needle attached to a 1 mL syringe. Collected samples were immediately aliquoted into sterile polypropylene tubes and cryopreserved at −80 °C pending biomarker analysis.

Quantification of 10 cytokines was performed using a flow cytometer (EASYCELL 206A1) with the CBA technique. Cytokine standards were reconstituted in 0.5 mL assay buffer provided by manufacturer, and equilibrated at room temperature (25℃) for 15 minutes. Serial dilutions (1:2.5 concentration gradient) were prepared using assay buffer to generate 8 standard calibrators.

Capture beads were prepared by combining 2 μL of each bead subset per sample with 35 μL bead diluent per sample in a single reaction tube. Similarly, detection antibodies were prepared by mixing 2 μL of each antibody per sample with 35 μL antibody diluent per sample.

Aqueous humor samples were volume-adjusted with assay buffer to meet the minimum detection volume requirements, followed by addition of matched volumes of capture bead mixture and detection antibody mixture. The reaction complexes were incubated in darkness under orbital shaking (18°C-25°C) for 2.5 hours.

Post-incubation, magnetic separation was performed to remove supernatant. Beads were washed twice with 200 μL wash buffer through sequential magnetic separation cycles, followed by resuspension in 200 μL fresh wash buffer. Flow cytometry analysis was performed immediately following instrument calibration using manufacturer-defined photomultiplier tube voltage settings.

### Covariates

Demographic data were obtained from self-reported questionnaires, including age, gender, duration of diabetes, the duration of ophthalmopathy, and history of hypertension and hyperlipidemia. The HbA1c, intraocular pressure, and affected eyes were collected from examination center system.

### Statistical analysis

All analyses were performed using the R.3.5 software. If the continuous data conformed to a normal distribution, they were expressed as the mean and standard deviation (*X*±*SD*), and a *t* test was conducted. The non-normal distribution continuous data were expressed as median (25th percentiles, 75th percentiles) [*M* (*P*_25_, *P*_75_)]. Count data were expressed as frequencies and percentages (*n,* %). This study employed a RCS model to assess the potential nonlinear relationship between the continuous variable HbA1c and inflammatory factors. Using RCS analysis, we set the knots for HbA1c based on its distribution percentiles, fitted a series of cubic polynomial functions at these knots, and constrained the ends with nonlinear functions to ensure stable extrapolation. To verify the impact of the number of konts on the model outcomes, we conducted a sensitivity analysis. Specifically, we re-fitted the RCS model with three to seven knots and compared the likelihood ratio test values across different knot numbers to confirm the goodness of fit of the RCS model. A lower Akaike information criterion (AIC) value indicated better model-data fit, and the model with the smallest AIC value was selected while considering the simplicity of the model. The model adjusted for age, gender, affected eye, the duration of ophthalmopathy, the duration of diabetes, hypertension, hyperlipoidemia, and intraocular pressure. Additionally, the Variance Inflation Factor (VIF) was employed to assess multicollinearity among parameters, with variables having a VIF greater than 5 being eliminated. Beta coefficients (β) with 95% confidence interval (CI) were reported as the measure of inflammatory factors. *p* < 0.05 indicated that the difference was statistically significant.

## Results

### Baseline characteristics

**Fig. 1:**
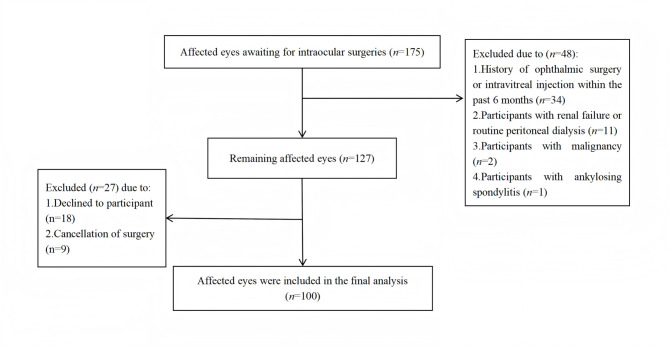
Participants recruited flow diagram

Between February and March 2025, a total of 138 participants were recruited, who had 175 affected eyes. In this study, we excluded participants who had undergone eye surgery within the past six months, suffered from renal failure, were on routine peritoneal dialysis, had malignancy, or had ankylosing spondylitis. 27 patients were excluded due to declined to participant or surgical cancellation. Ultimately, we successfully obtained aqueous humor samples from 100 eyes of 100 participants. The process of patient enrollment was shown in Fig. 1. The baseline characteristics of these participants were summarized in Table 1, and the levels of the 10 cytokines in their aqueous humor were listed in Table 2.

**Table 1:** Demographic information (*n* = 100 affected eyes from 100 participants)

Characteristic	Values
Age (year, *X*±*SD*)	58.2 ± 10.0
Gender (*n*, %)	
Male	56 (56.0%)
Female	44 (44.0%)
Duration of diabetes [year, *M* (*P*_25_, *P*_75_)]	15.0 (10.0, 23.3)
Duration of ophthalmopathy [year, *M* (*P*_25_, *P*_75_)]	0.25 (0.1, 1.0)
HbA1c (%, *X*±*SD*)	7.9 ± 1.5
Hypertension (*n*, %)	
Yes	94 (94.0%)
No	6 (6.0%)
Hyperlipidemia (*n*, %)	
Yes	12 (12.0%)
No	88 (88.0%)
Intraocular pressure [mmHg, *M* (*P*_25_, *P*_75_)]	14.7 (13.0, 16.0)
Affected eye (*n*, %)	
OD	61 (61.0%)
OS	39 (39.0%)

**Table 2:** Cytokine levels in aqueous humor sample (*n* = 100)

Aqueous cytokine	*M (P*_*25*_, *P*_*75*_)
IL-6 (pg/ml)	70.5 (32.3, 139.3)
IL-8 (pg/ml)	51.1 (24.7, 121.1)
MCP-1 (pg/ml)	1349.5 (662.7, 2387.2)
ICAM-1 (pg/ml)	938.5 (538.8, 1782.7)
CD106 (pg/ml)	3632.9 (2143.6, 8112.3)
VEGF (pg/ml)	128.0 (48.6, 500.7)
CXCL10 (pg/ml)	149.0 (57.6, 261.2)
G-CSF (pg/ml)	0 (0, 0.6)
IFN-γ (pg/ml)	0.1 (0, 0.4)
IL-10 (pg/ml)	0.4 (0, 1.1)

### Dose-response relationship between IL-6 and HbA1c

**Fig. 2:**
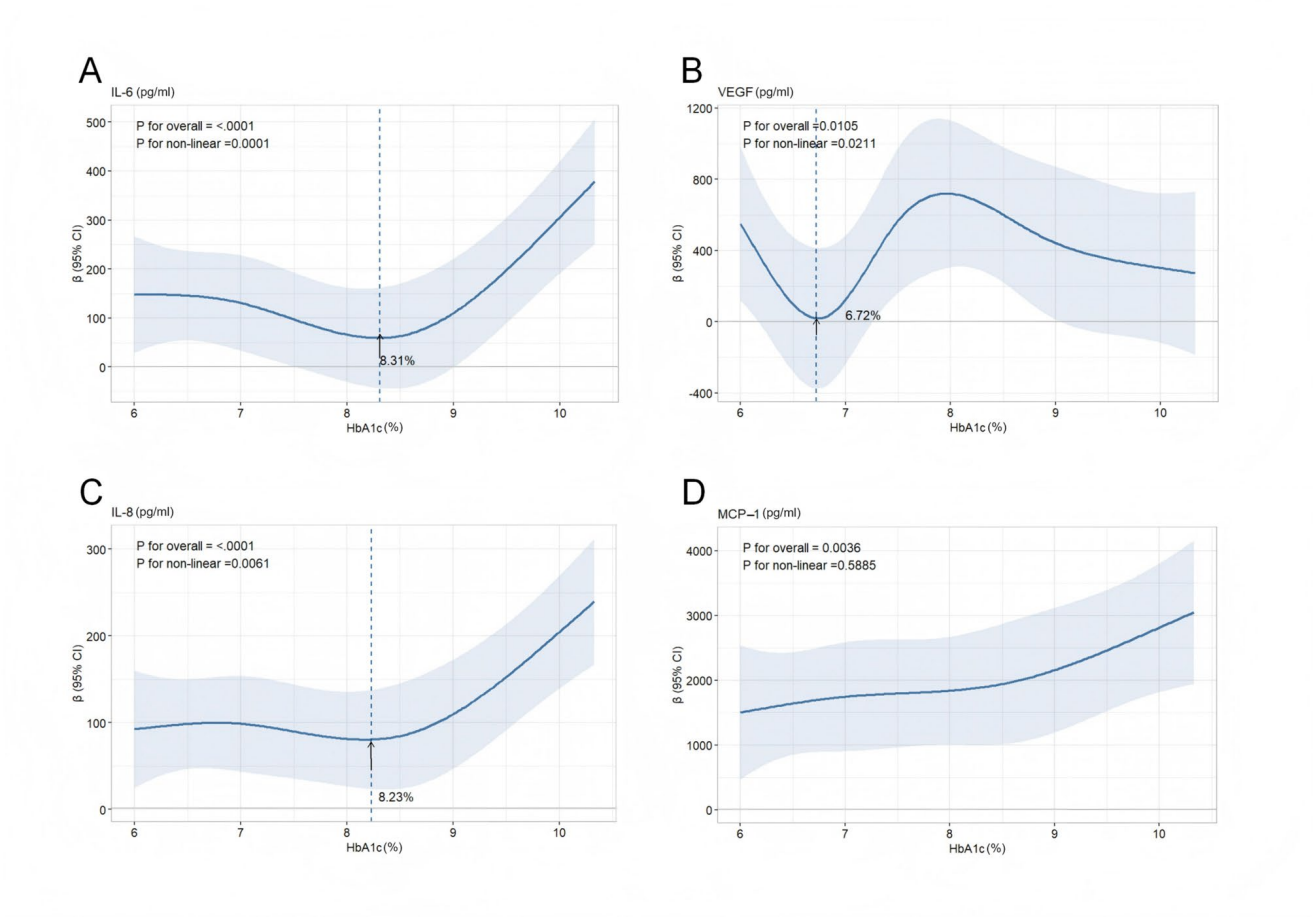
RCS for IL-6, VEGF, IL-8, and MCP-1 by HbA1c after covariate adjustment. Note: Heavy central line represented the trend with shaded ribbons denoting 95% CI. The position indicated by the arrow represents the turning point of the trend; The value shown by the arrow indicates the HbA1c concentration at the trend reversal. The models were adjusted for age, gender, duration of diabetes, the duration of ophthalmopathy, hypertension, hyperlipidemia, intraocular pressure, and affected eyes

The model achieved the best fit when the number of knots was set to four (with minimum AIC = 831.4). The VIF values of all covariates were < 5 (maximum VIF = 1.608), indicating that the model did not have significant collinearity issues. The RCS results showed an approximate U-shaped curve relationship between IL-6 and HbA1c (statistically significant nonlinear relationship, with a P-value of 0.0001). The inflection point occurred at an HbA1c level of 8.31%. Specifically, when the concentration of HbA1c was below 8.31%, we found a negative correlation between HbA1c and IL-6, meaning that as the level of HbA1c decreased, the level of IL-6 increased; When the HbA1c concentration was higher than 8.31%, we found a positive correlation between HbA1c and IL-6, that was, as the HbA1c level increased, the IL-6 level also increased (see Fig. 2A).

### Dose-response relationship between VEGF and HbA1c

Modeling was conducted using RCS of five knots (with minimum AIC of 992.9), and all covariates were adjusted (maximum VIF = 1.624). The relationship between VEGF and HbA1c presented a U-shaped curve (*P* for non-linear = 0.0211, see Fig. 2B). The turning point occurred when HbA1c was 6.72%. When the HbA1c concentration was below 6.72%, the IL-6 level increased as the HbA1c level decreased; When the HbA1c concentration was higher than 6.72%, as the HbA1c level increased, the IL-6 level increased accordingly.

### Dose-response relationship between IL-8 and HbA1c

When the knots was four, the model fitted best (AIC = 759.8). There was no multicollinearity between covariates (maximum VIF = 1.607). A J-shaped curve was observed in the relationship between IL-8 and HbA1c (statistically significant nonlinear association, *p* = 0.0061). Initially, IL-8 levels showed a relatively flat trend with increasing HbA1c. However, when HbA1c exceeded 8.23%, IL-8 exhibited a significant upward trend (Fig. 2C).

### Dose-response relationship between MCP-1 and HbA1c

The knots were set to four (with minimum AIC of 992.9), and the VIF values of all covariates were less than 5 (maximum VIF = 1.607). We observed a linear relationship between HbA1c and MCP-1, as shown in Fig. 2D (*P* for overall = 0.0036, *P* for non-linear = 0.5885). MCP-1 levels increased progressively with rising HbA1c.

### Other results

However, no significant differences were found between other inflammatory cytokines and HbA1c. More detailed results of sensitivity analysis and collinearity analysis were attached in the supplementary materials (for details, see Additional file 1).

## Discussion

This study revealed non-linear associations between HbA1c levels and IL-6, IL-8, and VEGF in PDR patients. By applying the RCS models, we identified U-shaped or J-shaped dose-response curves for these cytokines, with inflection points at HbA1c levels of 8.31%, 8.23%, and 6.72%, respectively. Additionally, we found a linear positive correlation between HbA1c levels and MCP-1 in PDR patients. These findings highlighted the complex interplay between chronic hyperglycemia, intraocular inflammation, and the occurrence of PDR, and underscore the importance of effective glycemic control.

Our study demonstrated a U-shaped relationship between HbA1c and VEGF, with the lowest aqueous humor VEGF concentration observed around an HbA1c level of 6.72%. Baharivand’s study demonstrated that PDR patients with well-controlled blood glucose (HbA1c < 7%) exhibited significantly lower serum and vitreous fluid VEGF levels [24], which is consistent with our research findings. However, when HbA1c levels were below 6.72%, aqueous humor VEGF concentrations increased with decreasing HbA1c levels. Feldman-Billard et al. hypothesized that in ischemic retinas, VEGF expression would be suppressed under sustained hyperglycemic conditions but would rebound upon glucose normalization [25], potentially mediated by insulin-induced HIF-1α activation in retinal epithelial cells [26].

The U-shaped and J-shaped relationships between HbA1c and IL-6/IL-8 suggestted the existence of two distinct biological pathways influenced by extreme glycemic levels. When HbA1c levels were below approximately 8.2%, the observed negative correlation reflected compensatory anti-inflammatory mechanisms activated during early or moderate hyperglycemia. For instance, transient hyperglycemia can induce adaptive responses such as increased production of antioxidants (e.g., glutathione) or upregulation of anti-inflammatory cytokines like IL-10, which may temporarily suppress pro-inflammatory mediators like IL-6 and IL-8 [27]. However, as HbA1c levels exceed this threshold, sustained hyperglycemia is likely to overwhelm these defenses, exacerbating oxidative stress, mitochondrial dysfunction, and accumulation of advanced glycation end-products (AGE)—all potent drivers of inflammation [28]. Notably, the interaction of AGE receptors activates nuclear factor kappa B, a master regulator of IL-6 and IL-8 transcription [29]. This biphasic response aligns with previous studies demonstrating that mild hyperglycemia may temporarily enhance cellular stress resistance, whereas chronic exposure precipitates irreversible damage [30].

Our study demonstrated a linear positive correlation between HbA1c and MCP-1. There was a significant and strong correlation between aqueous humor glucose concentration and blood glucose levels, with poor glycemic control leading to increased anterior chamber glucose permeability [31]. High glucose accelerates MCP-1 production in vascular endothelial cells via the P38 mitogen-activated protein kinase (P38 MAPK) pathway [32]. Significant correlations were observed between serum HbA1c and MCP-1 levels (*r* = 0.18, *p* = 0.05) [33]. Notably, a positive association between HbA1c and MCP-1 was evident in diabetic patients regardless of retinopathy status [34]. These results align with our research findings.

Furthermore, no significant associations were found between HbA1c and other cytokines (such as ICAM-1, CD106 or IP-10), which may reflect pathway-specific regulatory mechanisms. These observations highlighted the heterogeneity of cytokine networks involved in the pathogenesis of DR and suggest that the impact of HbA1c varies across different inflammatory pathways. In contrast, Mroczek’s study demonstrated that elevated vitreous CD106/VCAM-1 levels were associated with HbA1c concentration, which may be attributed to the heterogeneity of the study population [patients receiving intensive insulin therapy, with HbA1c (%) values of 9.21 ± 2.17] [35]. This discrepancy warrants further investigation.

Overall, our study demonstrated that serum HbA1c levels in PDR patients were correlated with aqueous humor levels of VEGF, IL-6, IL-8, and MCP-1. A meta-analysis [36] revealed that elevated levels of VEGF, IL-6, and MCP-1 in aqueous humor are associated with DR risk, while VEGF, IL-6, IL-8, and MCP-1 correlate with diabetic macular edema (DME) risk. These biomarkers may serve as potential predictive factors or therapeutic targets for DR/DME. Therefore, glycemic control may slow the progression of DR by modulating these biomarkers. However, in NPDR, the levels of most inflammatory factors (IL-6, IL-8, MCP-1, VEGF, and IP-10) were significantly lower than those in our study; no significant correlation was observed between IL-6, IL-8, IP-10, MCP-1, or VEGF and glycemic parameters [37]. The observed differences may be attributed to PDR involving a cascade of pathological changes induced by chronic hyperglycemia, including progressive retinal ischemia and hypoxia, aberrant neovascularization, VEGF-driven hyperpermeability, and enhanced inflammatory responses.

Our findings further emphasized the need for individualized HbA1c targets for patients in the management of DR. According to the research results, we recommend maintaining HbA1c levels between 6.5% and 7.0% for PDR patients, which aligns with the upper limit of the target range ( < 7%) recommended by the International Diabetes Federation [38]. However, in our study, most of participants had HbA1c levels above this threshold (mean HbA1c of 7.9 ± 1.5%). This discrepancy highlights the challenge of achieving tight glycemic control in the real world, especially in the older or long-term diabetic patients who were overrepresented in our sample (mean age 58.2 ± 10.0 years; median duration of diabetes 15.9 years). However, excessive intensification of glycemic control may also increase the risk of systemic complications. The ACCORD trial demonstrated that intensive glycemic control (HbA1c < 6.5%) increased mortality risk without reducing microvascular complications. However, on the basis of blood pressure control, an intensified glycemic control strategy reduced all-cause mortality risk [39]. In addition, older patients with multiple comorbidities may be more concerned about avoiding hypoglycemia [40], as hypoglycemia can lead to acute health risks, such as syncope or cardiac events, which could be a more immediate threat to elderly patients. Therefore, personalized blood glucose control targets are crucial in the management of DR, and they should take into account the patient’s comorbidities, risk of hypoglycemia, and the duration of diabetes. Furthermore, our study shows a high prevalence of hypertension and hyperlipidemia, which further emphasizes the importance of comprehensive management for DR patients. Effective blood pressure and lipid control not only reduces the risk of cardiovascular diseases but also alleviates endothelial dysfunction, which may indirectly affect the expression and activity of cytokines, thereby influencing inflammatory responses and retinal health.

Ethnic variations in HbA1c levels are well-documented; for instance, African Americans exhibit higher HbA1c than Caucasians at equivalent glucose levels, potentially leading to overdiagnosis of hyperglycemia [41]. Similarly, genetic polymorphisms in inflammatory pathways (e.g., IL-6 promoter variants) may alter cytokine responses to hyperglycemia across ethnic groups [42]. The lack of population-specific HbA1c reference ranges for Chinese individuals poses a critical barrier to optimizing DR care. Considering ethnic differences, the specific HbA1c threshold of Chinese should be determined through large-scale longitudinal studies. Such data would enhance the precision of risk stratification and therapeutic interventions in this high-risk population.

Therefore, the management of DR patients requires a multidisciplinary team approach, including endocrinologists, cardiovascular experts, dietitians, and ophthalmologists, to jointly formulate treatment plans. Such an integrated management strategy helps optimize blood glucose control, reduce the risk of DR progression, while considering the overall health status and quality of life of the patient. In this way, we can provide customized treatment plans for each patient to maximize health benefits and minimize risks.

This study has several limitations. First, its cross-sectional design precludes causal inference; longitudinal studies are needed to determine whether HbA1c fluctuations directly drive cytokine changes or vice versa. Second, the sample size (*n* = 100 patients) limits statistical power to detect modest associations, particularly for cytokines with low detection rates (e.g., G-CSF, IFN-γ, IL-10). Third, aqueous humor sampling during intraocular surgery introduces selection bias, as participants likely represent advanced DR cases requiring surgical intervention. Future studies should include non-surgical cohorts with varying DR severity to validate these findings. Additionally, the study did not account for medications influencing cytokine levels, such as statins, poglycemic agents, and so on. Statins, widely used for hyperlipidemia, possess anti-inflammatory properties that may attenuate IL-6 and IL-8 production [43]. Similarly, some hypoglycemic agents may have modulatory effects on inflammation beyond their glucose-lowering activity [44, 45]. These confounding factors warrant further investigation.

In conclusion, our study identified HbA1c-dependent thresholds for intraocular inflammation in PDR. These findings advocated for a nuanced approach to glycemic management, balancing the risks of hyperglycemia-induced damage and hypoglycemia-related harm. Future research should prioritize longitudinal cohorts, and integrative analyses of systemic and ocular biomarkers to refine DR prevention strategies.

## Electronic supplementary material

Below is the link to the electronic supplementary material.

Supplementary Material 1

## Data Availability

The study data are included in the manuscript and the supplementary material. Other information can be obtained from the corresponding author.
