# Supplementary material for: The association between glycated hemoglobin and intraocular inflammatory factors in patients with proliferative diabetic retinopathy
Source: Int J Retina Vitreous. 2025 Oct 22;11:115. doi: 10.1186/s40942-025-00732-y (PMC12542134; doi:10.1186/s40942-025-00732-y)
Supplement: Supplementary file 1 — Supplementary Material 1 [file 40942_2025_732_MOESM1_ESM.pdf]

1.IL-6 with HbA1c

(1)Sensitivity analysis

```
> #敏感性分析
> print(aic_summary)
                   Model Knots      AIC
knot4.d.f.  knot4.d.f.     4 831.3803
knot5.d.f.  knot5.d.f.     5 832.5978
knot6.d.f.  knot6.d.f.     6 832.9482
knot7.d.f.  knot7.d.f.     7 833.4515
knot3.d.f.  knot3.d.f.     3 834.0663
```

(2)Collinearity analysis

```
> #共线性分析
> vif(knot4)
        HbA1c         HbA1c'        HbA1c''        性别=女          年龄        眼别=左
    28.255164     604.449708     414.644970       1.242498       1.607700       1.104166
 眼病患病时长  糖尿病患病时长      高血压=无    高脂血症=无        术眼眼压
     1.210377       1.528832       1.341775       1.238241       1.259191
```

2.IL-8 with HbA1c

(1)Sensitivity analysis

```
> #敏感性分析
> print(aic_summary)
                   Model Knots      AIC
knot4.d.f.  knot4.d.f.     4 759.7708
knot6.d.f.  knot6.d.f.     6 759.9299
knot5.d.f.  knot5.d.f.     5 760.6684
knot7.d.f.  knot7.d.f.     7 761.0223
knot3.d.f.  knot3.d.f.     3 761.1034
```

(2)Collinearity analysis

```
> #共线性分析
> vif(knot4)
        HbA1c         HbA1c'        HbA1c''        性别=女          年龄        眼别=左
    28.255164     604.449708     414.644970       1.242498       1.607700       1.104166
 眼病患病时长  糖尿病患病时长      高血压=无    高脂血症=无        术眼眼压
     1.210377       1.528832       1.341775       1.238241       1.259191
```

3.MCP-1 with HbA1c

(1)Sensitivity analysis

```
> #敏感性分析
> print(aic_summary)
                   Model Knots      AIC
knot4.d.f.  knot4.d.f.     4 1103.663
knot5.d.f.  knot5.d.f.     5 1105.240
knot6.d.f.  knot6.d.f.     6 1107.086
knot7.d.f.  knot7.d.f.     7 1108.457
knot3.d.f.  knot3.d.f.     3 1115.711
```

(2)Collinearity analysis

```
> #共线性分析
> vif(knot4)
         HbA1c          HbA1c'         HbA1c''          性别=女            年龄           眼别=左
     28.255164      604.449708      414.644970        1.242498        1.607700        1.104166
  眼病患病时长  糖尿病患病时长        高血压=无      高脂血症=无          术眼眼压
      1.210377        1.528832        1.341775        1.238241        1.259191
```

4.ICAM-1 with HbA1c

(1) RCS for ICAM-1 and HbA1c

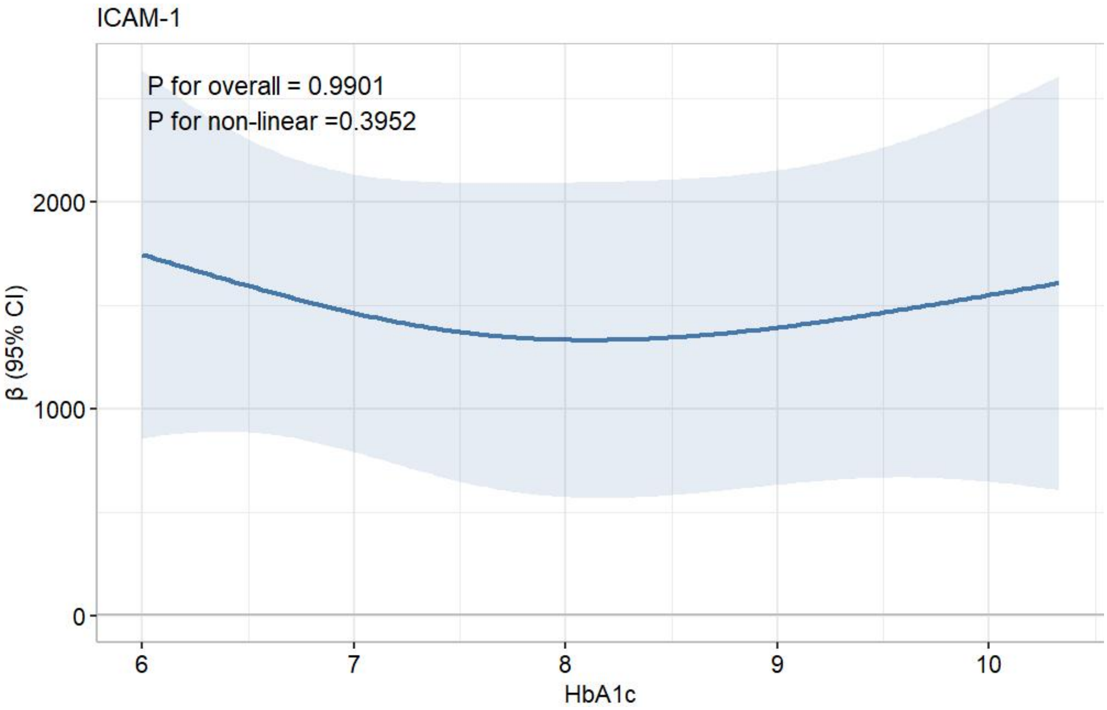

(2) Sensitivity analysis

```
> #敏感性分析
> print(aic_summary)
                Model Knots      AIC
knot3.d.f. knot3.d.f.     3 1091.087
knot4.d.f. knot4.d.f.     4 1093.070
knot7.d.f. knot7.d.f.     7 1093.191
knot6.d.f. knot6.d.f.     6 1093.648
knot5.d.f. knot5.d.f.     5 1094.710
```

(3) Collinearity analysis

```
> #共线性分析
> vif(knot3)
         HbA1c          HbA1c'         性别=女            年龄         眼别=左    眼病患病时长
     10.085858       10.034236        1.202633        1.607049        1.104009        1.193736
糖尿病患病时长        高血压=无      高脂血症=无        术眼眼压
      1.520347        1.331056        1.233397        1.232434
```

5.CD106 with HbA1c

(1) RCS for CD106 and HbA1c

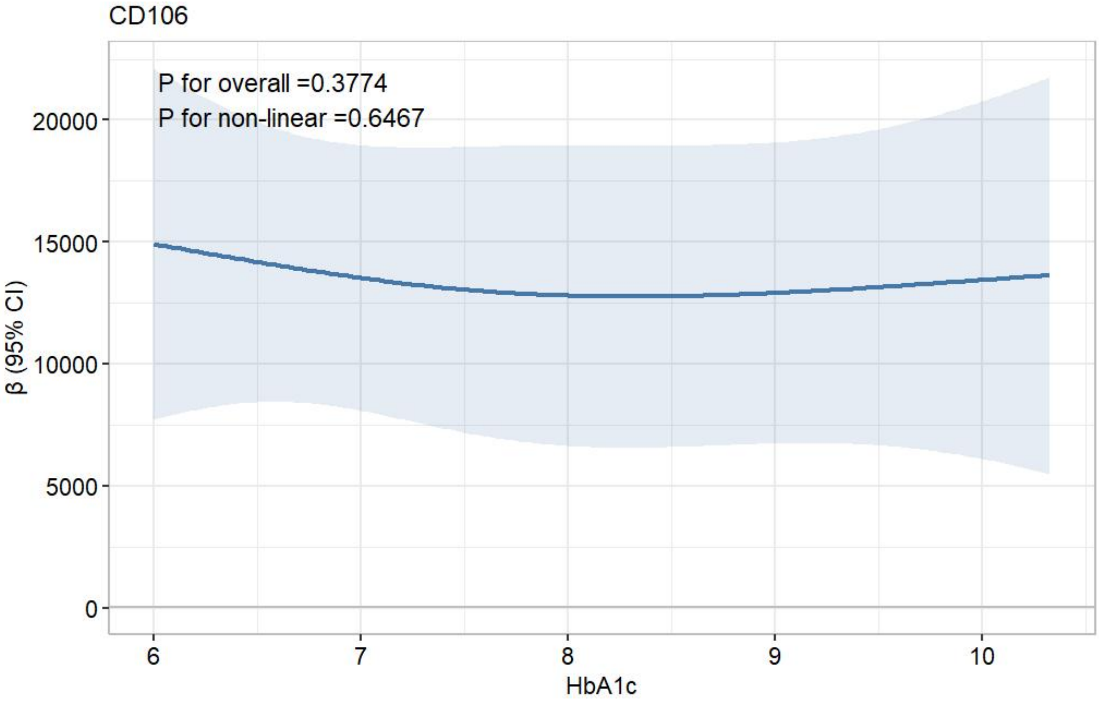

(2) Sensitivity analysis

```
> #敏感性分析
> print(aic_summary)
                      Model Knots      AIC
knot3.d.f.  knot3.d.f.      3 1354.892
knot4.d.f.  knot4.d.f.      4 1356.876
knot5.d.f.  knot5.d.f.      5 1357.761
knot6.d.f.  knot6.d.f.      6 1359.213
knot7.d.f.  knot7.d.f.      7 1360.095
```

(3) Collinearity analysis

```
> #共线性分析
> vif(knot3)
        HbA1c          HbA1c'          性别=女            年龄         眼别=左     眼病患病时长
    10.085858       10.034236        1.202633        1.607049        1.104009        1.193736
糖尿病患病时长        高血压=无       高脂血症=无          术眼眼压
     1.520347        1.331056        1.233397        1.232434
```

6.VEGF with HbA1c

(1) Sensitivity analysis

```
> #敏感性分析
> print(aic_summary)
                 Model Knots      AIC
knot5.d.f. knot5.d.f.     5 992.9253
knot6.d.f. knot6.d.f.     6 994.5619
knot7.d.f. knot7.d.f.     7 996.7993
knot4.d.f. knot4.d.f.     4 997.0844
knot3.d.f. knot3.d.f.     3 999.8997
```

(2) Collinearity analysis

```
> #共线性分析
> vif(knot5)
         HbA1c          HbA1c'         HbA1c''        HbA1c'''          性别=女            年龄
     58.613870     9261.492675    20879.039528     3165.457242        1.311988        1.624579
        眼别=左    眼病患病时长  糖尿病患病时长         高血压=无      高脂血症=无          术眼眼压
      1.105959        1.212655        1.530328        1.342024        1.253753        1.286573
```

7.CXCL10 with HbA1c

(1) RCS for CXCL10 and HbA1c

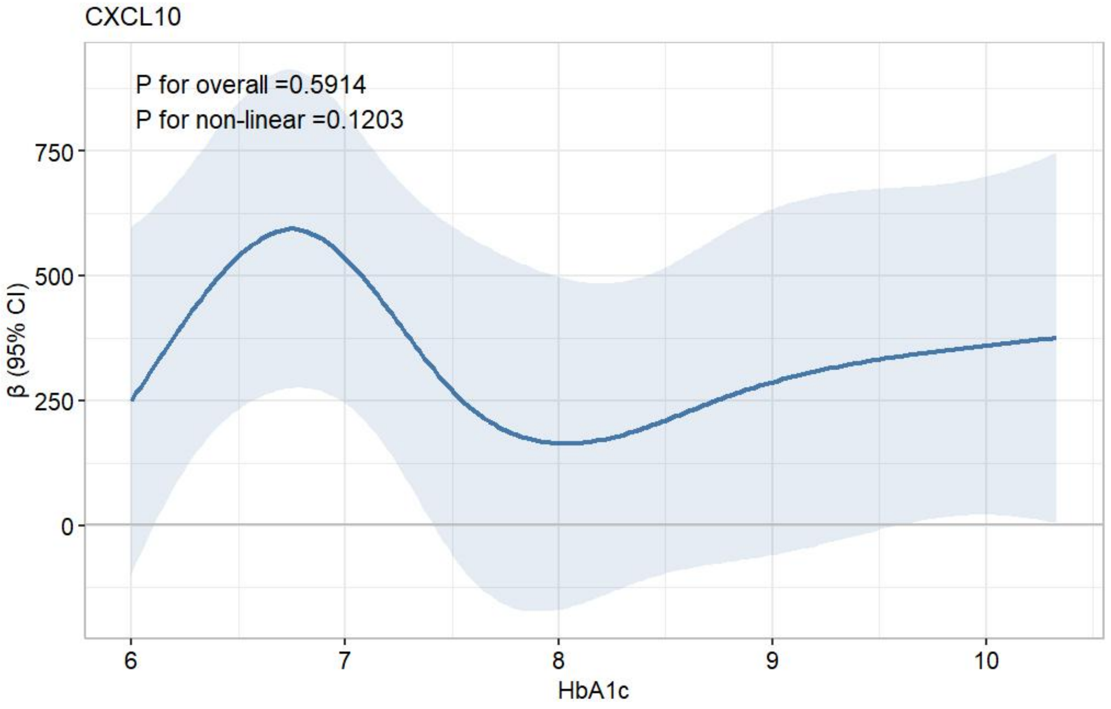

(2) Sensitivity analysis

```
> #敏感性分析
> print(aic_summary)
                Model Knots      AIC
knot5.d.f. knot5.d.f.     5 966.2935
knot4.d.f. knot4.d.f.     4 967.6335
knot6.d.f. knot6.d.f.     6 968.3227
knot3.d.f. knot3.d.f.     3 969.2143
knot7.d.f. knot7.d.f.     7 969.6676
```

(3) Collinearity analysis

```
> #共线性分析
> vif(knot5)
         HbA1c         HbA1c'        HbA1c''       HbA1c'''       性别=女         年龄
     58.613870    9261.492675   20879.039528    3165.457242      1.311988      1.624579
        眼别=左   眼病患病时长  糖尿病患病时长        高血压=无    高脂血症=无       术眼眼压
      1.105959       1.212655       1.530328       1.342024      1.253753      1.286573
```

8.G-CSF with HbA1c

(1) RCS for G-CSF and HbA1c

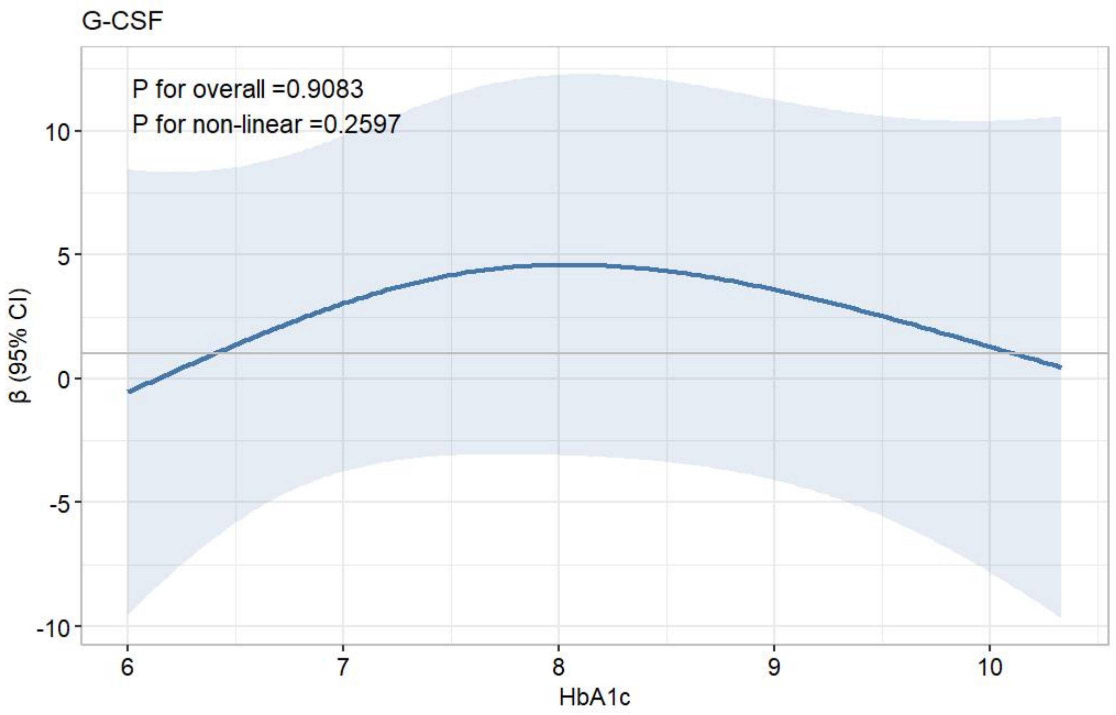

(2) Sensitivity analysis

```
> #敏感性分析
> print(aic_summary)
                  Model Knots      AIC
knot3.d.f.  knot3.d.f.      3 512.0432
knot7.d.f.  knot7.d.f.      7 512.4267
knot5.d.f.  knot5.d.f.      5 512.9472
knot4.d.f.  knot4.d.f.      4 514.1318
knot6.d.f.  knot6.d.f.      6 515.9802
```

(3) Collinearity analysis

```
> #共线性分析
> vif(knot3)
         HbA1c         HbA1c'        性别=女           年龄        眼别=左   眼病患病时长
     10.085858      10.034236       1.202633       1.607049      1.104009      1.193736
糖尿病患病时长      高血压=无    高脂血症=无       术眼眼压
      1.520347       1.331056       1.233397       1.232434
```

9.IFN-γ with HbA1c

(1) RCS for IFN-γ and HbA1c

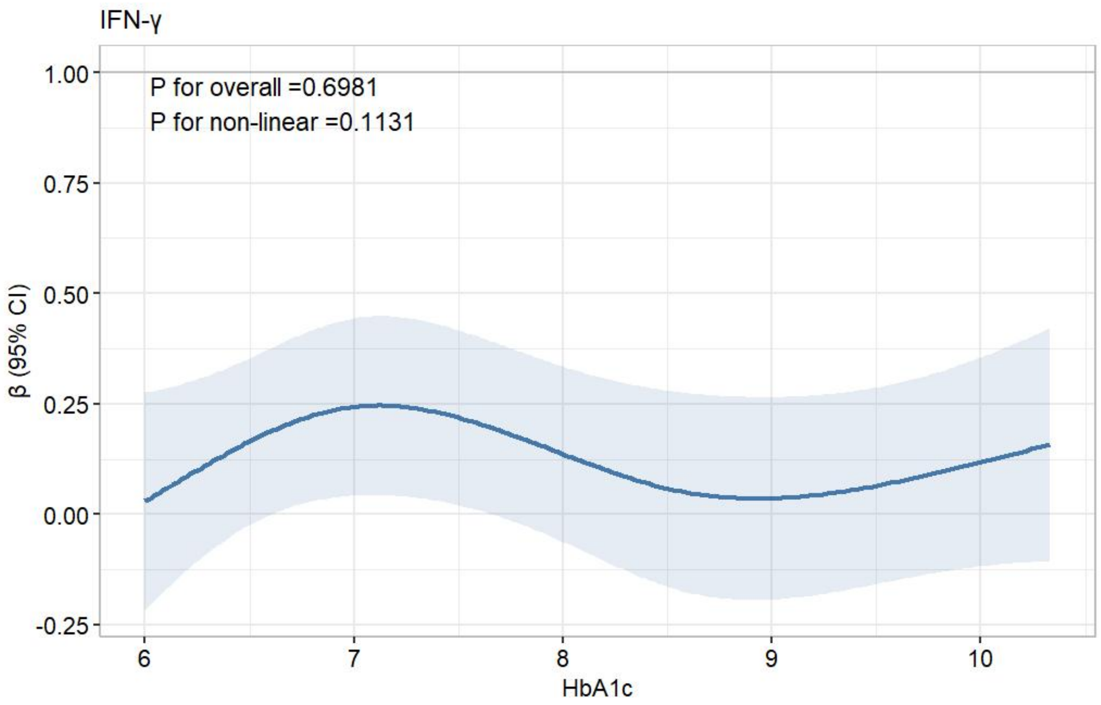

(2) Sensitivity analysis

```
> #敏感性分析
> print(aic_summary)
                  Model Knots      AIC
knot4.d.f. knot4.d.f.     4 52.51049
knot5.d.f. knot5.d.f.     5 54.33001
knot3.d.f. knot3.d.f.     3 55.50579
knot6.d.f. knot6.d.f.     6 56.26974
knot7.d.f. knot7.d.f.     7 57.19568
```

(3) Collinearity analysis

```
> #共线性分析
> vif(knot4)
         HbA1c          HbA1c'         HbA1c''          性别=女            年龄          眼别=左
     28.255164      604.449708      414.644970        1.242498        1.607700        1.104166
  眼病患病时长  糖尿病患病时长       高血压=无      高脂血症=无           术眼眼压
      1.210377        1.528832        1.341775        1.238241        1.259191
```

10.IL-10 with HbA1c

(1) RCS for IL-10 and HbA1c

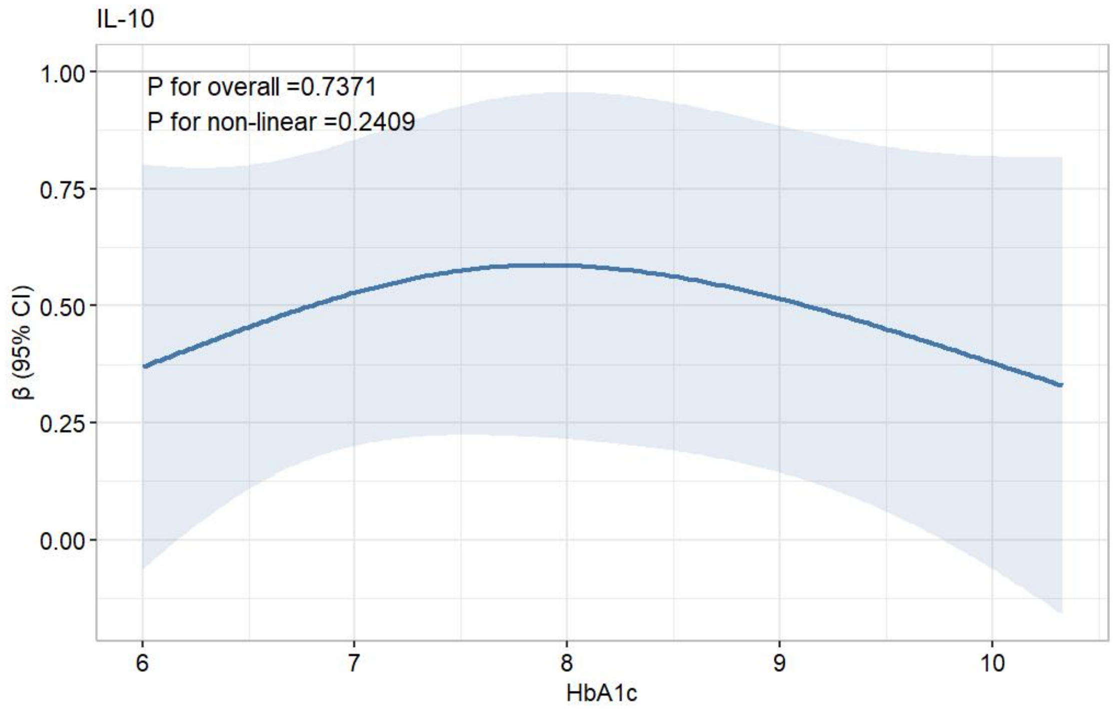

(2) Sensitivity analysis

```
> #敏感性分析
> print(aic_summary)
                 Model Knots      AIC
knot3.d.f. knot3.d.f.     3 130.3157
knot4.d.f. knot4.d.f.     4 132.1199
knot5.d.f. knot5.d.f.     5 133.4869
knot7.d.f. knot7.d.f.     7 134.2757
knot6.d.f. knot6.d.f.     6 135.4204
```

(3) Collinearity analysis

```
> #共线性分析
> vif(knot3)
      HbA1c        HbA1c'        性别=女          年龄        眼别=左    眼病患病时长
  10.085858     10.034236      1.202633      1.607049      1.104009      1.193736
糖尿病患病时长       高血压=无     高脂血症=无         术眼眼压
   1.520347      1.331056      1.233397      1.232434
```
